# A Suicide Prevention Digital Technology for Individuals Experiencing an Acute Suicide Crisis in Emergency Departments: Naturalistic Observational Study of Real-World Acceptability, Feasibility, and Safety

**DOI:** 10.2196/52293

**Published:** 2024-09-16

**Authors:** Linda A Dimeff, Kelly Koerner, Kandi Heard, Allison K Ruork, Angela Kelley-Brimer, Suzanne T Witterholt, Mary Beth Lardizabal, Joseph R Clubb, Julie McComish, Arpan Waghray, Roger Dowdy, Sara Asad-Pursley, Maria Ilac, Hannah Lawrence, Frank Zhou, Blair Beadnell

**Affiliations:** 1 Evidence-Based Practice Institute Beaverton, OR United States; 2 Evidence-Based Practice Institute Seattle, WA United States; 3 Mental Health and Addiction Clinical Service Line Allina Health Minneapolis, MN United States; 4 Providence's Well Being Trust Providence Renton, WA United States; 5 Evaluation Specialists Carlsbad, CA United States

**Keywords:** suicide, emergency department, ED, digital technology, suicide prevention best practices, individual, particular, suicide prevention, evidence-based intervention, Emergency department, hospital, vulnerable population, Jaspr Health, psychiatric, psychiatrist, care, safety

## Abstract

**Background:**

Emergency departments (EDs) are the front line in providing suicide care. Expert consensus recommends the delivery of several suicide prevention evidence-based interventions for individuals with acute suicidal ideation in the ED. ED personnel demands and staff shortages compromise delivery and contribute to long wait times and unnecessary hospitalization. Digital technologies can play an important role in helping EDs deliver suicide care without placing further demands on the care team if their use is safe to patients in a routine care context.

**Objective:**

This study evaluates the safety and effectiveness of an evidence-based digital technology (Jaspr Health) designed for persons with acute suicidal ideation seeking psychiatric crisis ED services when used as part of routine ED-based suicide care. This study deployed Jaspr Health for real-world use in 2 large health care systems in the United States and aimed to evaluate (1) how and whether Jaspr Health could be safely and effectively used outside the context of a researcher-facilitated clinical trial, and (2) that Jaspr’s use would be associated with improved patient agitation and distress.

**Methods:**

Under the auspices of a nonsignificant risk device study, ED patients with acute suicidal ideation (N=962) from 2 health care systems representing 10 EDs received access to Jaspr Health as part of their routine suicide care. Primary outcome measures included how many eligible patients were assigned Jaspr Health, which modules were assigned and completed, and finally, the number of adverse events reported by patients or by medical staff. Secondary outcome measures were patient agitation, distress, and satisfaction.

**Results:**

The most frequent modules assigned were Comfort and Skills (98% of users; n=942) and lethal means assessment (90% of patient users; n=870). Patient task completion rates for all modules ranged from 51% to 79%. No adverse events were reported, suggesting that digital technologies can be safely used for people seeking ED-based psychiatric services. Statistically significant (*P*<.001) reductions in agitation and distress were reported after using the app. Average patient satisfaction ratings by site were 7.81 (SD 2.22) and 7.10 (SD 2.65), with 88.8% (n=325) and 84% (n=90) of patients recommending the app to others.

**Conclusions:**

Digital technologies such as Jaspr Health may be safely and effectively integrated into existing workflows to help deliver evidence-based suicide care in EDs. These findings hold promise for the use of digital technologies in delivering evidence-based care to other vulnerable populations in complex environments.

## Introduction

Globally, suicide remains a significant public health problem accounting for approximately 700,000 deaths annually [1] and is the fourth leading cause of death for people aged 15-29 years worldwide [2]. In the United States, suicide is the ninth leading cause of death for individuals aged 10-64 years, and the second leading cause of death for individuals aged 10-14 and 25-34 years [3]. In 2021, a total of 47,646 suicides were recorded in the United States—a 4% increase from the previous year [4]. In addition to the lives lost, far more people in the United States seriously think about (12.2 million), make a plan for (3.2 million), or attempt (1.2 million) suicide annually [5]. The impact of suicide and suicidal behaviors are far greater, as families, coworkers, and communities also suffer from the loss of their loved one to suicide or from seeking capable help [6-9].

In the United States, emergency departments (EDs) are the front line for suicide prevention, providing psychiatric crisis care for those with acute suicidal ideation [10-12]. Nearly 650,000 people are evaluated for suicide attempts in US EDs annually [13-15]; approximately 1.4 million ED visits annually are for suicidal ideation or deliberate nonsuicidal self-injury [16-18]. Recognizing the unique challenges in treating suicidality in the ED, The Joint Commission (TJC) recommends targeting three domains: (1) address patients’ needs (eg, rapid nonsedating treatment of agitation, mitigating stressors, initiating active treatment), (2) support ED staff (eg, in reducing patient’s distress; assessing patient’s progress over time to determine readiness for discharge), and (3) improve the environment (eg, avoid psychiatric hospitalization) [19].

Unfortunately, ED-based behavioral health emergencies continue to rise in number, with 6%-10% of all ED patients presenting with a psychiatric emergency [20,21]. This rise in the need for psychiatric care produces an increased burden on staff [22-26]. Furthermore, behavioral health emergencies require approximately 42% more time than nonpsychiatric visits and result in twice the rate of inpatient admission [27], thus, contributing to the problem of boarding as patients can remain in the ED for up to 4 hours after a decision is made to admit them [19,22]. The burden of treating patients with acute suicidal ideation in the ED cannot be overstated [19,23]—longer ED stays affect patients and providers, and cost the system an estimated US $2264 per patient [19,25,28]. Such challenges make it difficult for many EDs to comply with TJC recommendations [29] and there are few tools to help ensure that EDs meet these targets, without placing increased demands on under-resourced settings (eg, additional staffing, training, oversight is often near impossible in more rural EDs). Thus, novel approaches are needed to address the significant public health impact of suicide.

Digital technologies can efficiently and effectively deliver evidence-based interventions, and are highly scalable, [30-32] critical in the case of ED-based suicide care. In addition, digital interventions also appear to facilitate more complete patient disclosure [33]. Moreover, digital interventions can deliver helpful tools to patients, and simultaneously ease the problem of patient boarding by facilitating the collection of necessary information for discharge (eg, safety plans), while patients are waiting for providers [34]. Streamlining of data collection and delivery of helpful tools without the need of additional personnel may be particularly helpful in systems with limited behavioral health resources (eg, EDs in more rural areas). What is needed is a study on the deployment of a digital intervention to support suicide care in EDs, specifically examining how such a technology would be used by staff and its impact on patient safety and stabilization.

### Background on the Intervention

To help address the needs of providers and improve the quality of care for individuals in acute suicide crisis, we developed a tablet-based crisis app (Jaspr Health; “Jaspr”) for people experiencing suicidality and seeking suicide crisis care in EDs. Jaspr was developed in consultation with suicide experts, with a goal of translating standards of clinical care for suicide common across intervention models (eg, risk assessment, safety planning, lethal means assessment, and management) [35]. Jaspr helps medical care teams deliver these expert-recommended evidence-based suicide care [36-39]. Specifically, Jaspr digitizes a comprehensive suicide risk assessment, assessment of lethal means, and building of a crisis stability plan for review by the patient’s ED provider, all of which becomes part of the patient’s health record via integration with electronic health record software (eg, Epic). Furthermore, it strongly aligns with all 3 domains recommended by TJC: patients can view videos of people with lived experience sharing their wisdom and teaching behavioral skills intended to reduce patient agitation and distress; Jaspr-at-Home, a companion app, bundles Jaspr for home use during the high-risk postdischarge period. In addition, it bundles these evidence-based suicide care practices in 1 system for ease of delivery. Results from a small pilot randomized controlled trial (RCT) comparing Jaspr (n=14) to care-as-usual (CAU; n=17) in ED adults with acute suicidal ideation, demonstrated its superiority to CAU. Jaspr participants were significantly more likely to receive evidence-based procedures in the ED, reported significant decreases in agitation and distress over time, and reported significant increases in their capacity to cope with suicidal distress. Jaspr participants also reported higher overall satisfaction with their ED experience (approached significance: *P*=.06; large effect: Cohen *d*=.8), with 100% of users recommending its use [37]. (The ED-based study was discontinued due to the COVID-19 pandemic, which began impacting EDs 6 weeks after the RCT’s start). While RCT findings were encouraging, large-scale data without a research team driving participation was necessary to understand how Jaspr would be implemented in real-world settings.

### This Study

This study deployed Jaspr for real-world use in 2 large health care systems in the United States, and aimed to evaluate (1) how and whether Jaspr could be safely and effectively used outside of the context of a researcher-facilitated clinical trial, and assuming that with delivery of evidence-based care comes improved patient stabilization and satisfaction (2) that Jaspr’s use would be associated with improved patient outcomes, consistent with the pilot RCT [37]. To evaluate Aim 1, exploratory data were collected on how medical staff deployed Jaspr, including (1a) frequency of module assignment and (1b) completion rates for individual Jaspr modules, as well as (1c) presence of adverse events (AEs) related to study participation, including use of Jaspr. For Aim 2, hypotheses were consistent with the prior RCT, specifically, (2a) that Jaspr use would produce significant reductions in self-reported agitation and distress and (2b) high care satisfaction ratings.

## Methods

### Setting

We implemented Jaspr at 10 sites serving patients with acute suicidal ideation throughout 2 geographically diverse health care systems, Allina Health (“Allina”) and Providence. Allina includes 13 hospitals located throughout Minnesota, with its largest campuses located near the high-density cities of Minneapolis and St. Paul. As part of the initial Allina implementation, sites were selected by each system’s behavioral health leadership. Criteria for ED selection included: high volume of behavioral health emergencies in the ED, high need for behavioral health resources, strong ED leadership with prior success launching other innovative programs, and a request to participate. With 51 hospitals in its system, Providence serves patients across 7 states, including Alaska, California, Montana, New Mexico, Oregon, Texas, and Washington. Implementation at Providence was based on testing Jaspr in a wide variety of EDs, that varied by setting (eg, rural vs urban), workflow (eg, with mental health clinicians or rapid response teams vs delivery by nursing or other staff), and resources. Data from these settings were collected between January 17, 2022, and December 21, 2022.

Each system assembled an implementation team to work collaboratively on their respective rollouts. Each team met weekly with Jaspr’s implementation team for several months in preparation for its use with patients. Topics addressed included: selection of EDs, sequencing of ED rollout, analyzing existing workflows for optimal insertion of Jaspr, selection of site leaders, eligibility criteria for Jaspr use, information technology’s review of Jaspr’s HIPAA (Health Insurance Portability and Accountability Act) compliance standards and other security measures, and review of institutional review board (IRB) approval and nonsignificant risk device study status. In total, 6 EDs from Allina (Mercy Hospital, Coon Rapids, Minnesota [MN]; Unity Hospital, Fridley, MN; Abbott Northwestern Hospital, Minneapolis, MN; United Hospital, St. Paul, MN; St. Francis Regional Medical Center, Shakopee, MN; Cambridge Medical Center; Cambridge, MN) and 4 from Providence (St. Mary’s Hospital, Walla Walla, WA; St. Patrick’s Hospital, Missoula, MT; Swedish Edmonds, Edmonds, WA; Portland Medical Center, Portland, OR) implemented Jaspr in their EDs.

### Ethical Considerations

All procedures were approved by Association for the Accreditation of Human Research Protection Programs, Inc–accredited Sterling IRB and each system’s IRB (Sterling IRB #9187, WCG IRB #1841871). External monitoring was provided by an independent Data Safety Monitoring Board comprising suicide experts. An IRB-approved consent document was displayed at the start of participation in Jaspr; participants could either affirm consent by pressing “I understand and agree to take part in this research” or could simply not press forward and return the tablet to staff. Use of Jaspr was not incentivized and there were no penalties or other changes to care for patients not participating. Study data were collected solely within the Jaspr environment, which was accessed via dedicated tablet and stored within the patient’s medical record. Deidentified data were stored in a HIPAA-compliant cloud-based server for analyses.

### Participants

In total, 706 Allina patients and 256 Providence patients (N=962) were assigned activities in Jaspr. Eligible participants were English-speaking individuals seeking psychiatric crisis services for suicidality in the ED, as well as individuals admitted to a psychiatric inpatient unit for suicidality (Allina). Age eligibility differed between the 2 systems: Allina participants were 18 years or older; Providence participants were 13 years or older with parent or legal guardian permission collected where required by state law for those under 18 years of age. Individuals who were acutely psychotic, severely agitated, or significantly impaired by alcohol or drugs were excluded from participating because they would be unable to provide informed consent, meaningfully engage with Jaspr, and because of safety concerns involving access to a tablet that could be weaponized.

### Procedures

Eligibility was determined by nurses or behavioral health specialists. Once deemed eligible, a medical provider introduced the patient to Jaspr and invited them to use the app. For those interested, the provider then activated the app and assigned specific Jaspr modules based on the patient’s needs. When first accessing Jaspr, patients were oriented to the purpose of the app and their rights: Jaspr use was voluntary and was intended to supplement their care, not replace treatment provided by their medical care team. Consenting patients then engaged in using their assigned Jaspr activities. Patients used the app for as long or little as they wished, while waiting to receive direct care from their care teams. Medical care teams were expected to review the patient’s answers to the comprehensive suicide assessment, lethal means assessment, and crisis stability plan as part of their own clinical assessment; they could edit the responses based on discussion with their patient. Patients wishing to access the app’s content at home could register for Jaspr-at-Home for continued use post discharge at no cost. Care teams were instructed to help patients with the Jaspr-at-Home app installation process when possible, and when not (eg, units that did not allow cell phones) provide instructions for setup in the patient’s discharge paperwork.

### Measures

#### Primary Outcomes (Aim 1): Assignment, Completion, and Safety

As our primary aim was to evaluate how and whether Jaspr was deployed in real-world settings, outcome measures included how many eligible patients were assigned Jaspr, which modules were assigned and completed, and finally, number of AEs reported within the app by patients or by medical staff. To ensure the ease of reporting AEs, patients could directly report an AE within the app itself or alert their medical team, who could also record AEs from within their own Jaspr portal. Patients and providers could also directly contact Jaspr researchers via phone or email. AEs could be reported at any point during app usage or post discharge through Jaspr-at-Home. The researchers were electronically alerted immediately upon report of an AE.

#### Secondary Outcomes (Aim 2): Patient Care and Satisfaction

Patients completed brief self-report measures within Jaspr to assess agitation, distress, and care satisfaction. In general, the current study replicated measures used in the RCT [37] where measures were selected for their brevity and simplicity for individuals with acute suicidal ideation. Two items from the *Safety and Imminent Distress Questionnaire*, a 4-item face-valid self-report survey based on Boudreaux’s *Keeping Myself Safe Subject Usability Survey* [40] were used to assess agitation and distress. Participants rated their feelings in the present using a 10-point scale. The following items were included: intensity of emotional distress (1=no distress; 10=highest distress ever felt) and the extent to which they felt calm or agitated (1=very calm; 10=very frustrated or agitated). One item from the 7-item *Emergency Room Patient Satisfaction Survey*, developed in consultation with the patient experience division of a large health care organization, was selected to assess overall satisfaction with their ED experience (1=worst experience; 5=best experience). One item involved providing rating of Jaspr (1=poor; 10=excellent). Finally, patients were asked if they would recommend Jaspr to others in their situation (yes/no).

### Intervention

Jaspr is intended to help ED health care professionals deliver evidence-based suicide care for individuals with acute suicidal ideation [37]. Patients are welcomed into the app via a video by suicide experts and an individual with lived experience. Patients are walked through several evidence-based practices in a conversational tone, including a risk assessment flow, safety and stability plan creation, lethal means assessment, and distress tolerance skills practices (these elements are based on the Collaborative Assessment and Management of Suicidality (CAMS) Suicide Status Interview (SSI, Sections A and B) [41,42], CAMS crisis stability plan and lethal means assessment (SSI, Section C), and behavioral skills training for managing emotional distress [43,44] respectively). In addition, there is content related to suicide prevention psychoeducation and an extensive library of videos by people with lived experience sharing their own recovery stories and generating hope. Psychoeducation videos span a range of topics including what to expect in the ED, when is inpatient hospitalization indicated, and common reasons why people feel suicidal. “Shared Stories” by people with lived experience include coping with shame, strategies for staying well, and messages of hope (“My Wish for You”). For a more thorough description of Jaspr, see Dimeff et al [37] and supplementary file.

Jaspr tools were bundled for provider ease in assigning them to patients based on need. Bundles included: Comprehensive Suicide Assessment, Safety Planning (crisis stabilization and lethal means assessment), Comfort and Skills (behavioral skills training), and Shared Stories. The election of bundles to assign was at the discretion of the care team and varied based on the patient's needs, the care the patient received before Jaspr, and the estimated time before the discharge or transfer to an inpatient floor. If patients were not assigned a bundle, they could not access it. However, all bundles included lethal means assessment (paired with the first bundle assigned). Additionally, if a user was assigned the Safety Planning bundle, they could access video content from Comfort and Skills. Due to TJC’s requirement that all patients with suicidal ideation receive the crisis stability plan before discharge, system leaders encouraged the Safety Planning bundle at a minimum.

### Statistical Analyses

Analyses focused on assignment and completion rates, pre- to post-intervention change, and satisfaction. All analyses were conducted separately for Allina and Providence patients to identify patterns across systems, and out of recognition that each system has its own workflows, policies, and procedures. Finally, paired sample *t* tests were conducted in SPSS (version 27; IBM Corp) [45] to compare pre- and posttest scores for agitation and distress. There was no *a priori* sample size requirement for analysis; the sample was based only on the total number of participants who provided data during the data collection window.

## Results

### Aim 1a: Assignment

In total, 706 Allina patients and 256 Providence patients (N=962) were assigned at least one Jaspr bundle. Across both systems, Comfort and Skills was by far the most assigned feature, with 98% of all users being assigned its content (n=942). Jaspr’s lethal means assessment was the second most commonly assigned feature, assigned to 90% of patient users (*n*=870). Overall, 86% were assigned to use Jaspr to build a crisis stability plan (n=830). The Comprehensive Suicide Assessment was the least assigned bundle, assigned to fewer than half of Jaspr users (n=470).

### Aim 1b: Completion

Assigned bundles were completed at moderately high rates. Across both health care systems, the Comprehensive Suicide Assessment had the highest rate of completion (74%; n=348), followed by lethal means assessment (68%; n=591), and then building a crisis stability plan (59%; n=489). Since there are numerous hours of Comfort and Skills available within Jaspr, there is no a priori determinant of completion. In total, 42.8% of Allina patients (n=302) and 29% of Providence patients (n=75) set up Jaspr-at-Home (Table 1).

**Table 1:** Assignment and completion rates of Jaspr’s modules per hospital system.

	Allina (n=706)				Providence (n=256)		
	Assigned	Completed	Completed (%)	Assigned		Completed	Completed (%)
CSA^a^	327	259	79	143		89	62
CSP^b^	625	384	61	205		105	51
Lethal Means	643	451	70	227		140	62
C&S^c^	689	—^d^	—	253		—	—

^a^CSA: Comprehensive Suicide Assessment.

^b^CSP: Crisis Safety Plan.

^c^C&S: Comfort and Skills.

^d^Not applicable.

### Aim 1c: AEs

Of the 962 ED patient users of Jaspr with acute suicidal ideation to date, there have been no serious AEs reported. Moreover, only one report of a potential AE was made, which after review by the Data Safety Monitoring Board was classified as a software “bug” requiring fixing, not an AE. Specifically, a provider noted that a patient was able to exit the Jaspr app and access the internet. No harm to the patient was reported and the bug was quickly addressed.

### Aim 2a: Treatment Outcomes

Statistically significant decreases in agitation and distress were observed across patients in both health care systems after using Jaspr (Table 2). Pre-post within-condition effect sizes at Allina were medium for agitation (*d*=0.46) and distress (*d*=0.56). At Providence, effect sizes were also medium for agitation (*d*=0.51) and distress (*d*=0.51).

**Table 2:** Paired sample t tests and means for pre- and postvisit distress and agitation levels among participants by hospital system.

		N	Pre, mean (SD)	Post, mean (SD)	*t* test (*df*)	Cohen *d*	Sig. (2-tailed)
**Distress**							
	Allina	366	5.46 (2.60)	4.35 (2.63)	10.69 (365)	0.56	<.001
	Providence	112	5.58 (2.45)	4.59 (2.53)	5.42 (111)	0.51	<.001
**Agitation**							
	Allina	366	4.63 (2.75)	3.65 (2.63)	8.75 (365)	0.46	<.001
	Providence	109	5.09 (2.84)	4.14 (2.52)	5.32 (108)	0.51	<.001

### Aim 2b: Satisfaction

High levels of satisfaction were reported by patients at both health care systems. Specifically, of those who provided satisfaction ratings, 88.8% of Allina patients (n=325) and 84% of Providence patients (n=90) recommended Jaspr using a binary true or false response. The average Jaspr satisfaction rating on the 10-point satisfaction survey was 7.81 (SD 2.22) for Allina patients and 7.10 (SD 2.65) for Providence patients. The average ED rating, scored on a 5-point scale, was 3.96 (SD 1.08) for Allina and 3.79 (SD 1.13) for Providence.

## Discussion

### Relevance and Findings

EDs remain the frontline in suicide prevention. For this reason, consensus-derived standards define ED care for persons with acute suicidal ideation seeking psychiatric crisis services, some of which are now regulatory requirements. Yet, few EDs are adequately equipped with the resources to deliver these important life-saving interventions. Jaspr was designed to help ED health care providers in delivering evidence-based suicide care at the point of need. Findings from a previous pilot RCT supported Jaspr’s feasibility and efficacy: in comparison to CAU, Jaspr significantly increased delivery of expert-recommended suicide care for patients with acute suicidal ideation in the ED, reduced agitation and distress, and increased capacity to cope with a suicide crisis. This study sought to extend these findings and determine its safety when used in real-world conditions, where the app’s use was integrated into EDs’ natural workflows and without the tightly controlled research procedures. Results suggest that Jaspr was regularly assigned to patients, who completed the various modules at moderately high rates. It was safe to use, associated with decreases in agitation and distress, and reported high satisfaction rates.

Across 10 EDs and 1 inpatient unit within 2 large health care systems, Jaspr was used by ED health care teams to aid their suicide care to 962 individuals with acute suicidal ideation seeking psychiatric crisis services. No AEs occurred, supporting the safety of the Jaspr crisis app in particular, and digital aids in general, for individuals with acute suicidal ideation, even in a highly complex, fast-paced ED. Consistent with our previous research, patients also experienced significant reductions in agitation and distress over the course of their use, a behavioral target encouraged by TJC. While the clinical significance of these specific changes is not clear, decreases in agitation and distress may improve ED outcomes for patients with suicidal ideation by decreasing the need for pharmacological interventions or restraint, and help facilitate other ED interventions (eg, staff assessment, treatment, and discharge planning) [11,46,47].

Of note is also how Jaspr was used by medical providers and patients. In both health care systems, providers were most likely to assign content that helped patients distract from their suicide crisis and learn new behavioral skills to better cope with negative emotions. This may account for the significant reductions in agitation and distress. The comprehensive suicide assessment was the least assigned, though it had the highest completion rate by patients when assigned. The low assignment may be because both health care systems routinely use a different instrument to assess suicidality (the Columbia Suicide Severity Rating Scale [48]) from the assessment instrument embedded in Jaspr (CAMS SSI). While overall usage was comparatively low, 348 patients—over a third of the total sample—fully completed an evidence-based suicide risk assessment. Other life-saving components were assigned at a high rate: lethal means assessment was assigned in 91% of all cases and safety planning in 86% of all cases. Even though only 68% fully completed the lethal means assessment and 59% built a crisis stability plan using Jaspr, a sizeable number of patients received and completed these important interventions to fidelity (n=591 and n=489, respectively). These results are particularly promising as crisis stability planning and lethal means assessment are practices recommended by TJC [49]. Future research to clarify under what conditions these modules were discontinued would be helpful in improving the intervention and ensuring delivery of recommended care. In addition, nearly all patients who provided ratings from both systems positively endorsed Jaspr (89% at Allina; 84% at Providence) and recommended it for others in the midst of a suicide crisis in the ED.

These data suggest that when used in conjunction with provider-led care, tools like Jaspr allow for more standardized delivery of evidence-based suicide care, particularly delivery of safety planning and lethal means assessment. Such standardization is beneficial for the patient, who is stabilized and can return home more readily. However, the system and payers also benefit, as it reduces costs, reduces the problems associated with boarding in the ED [19,22,25], saves providers time by gathering and documenting necessary information (standardized documentation also reduces the potential for lawsuits) all without requiring allocation of additional personnel. Future research would benefit from a more granular examination of the benefits of tools like Jaspr, including the aspects of the intervention that are most helpful, the relation to patient outcomes, in both the short (eg, agitation, distress) and long terms (eg, lost productivity, subsequent hospitalization), as well as system costs, and discharge timeline. However, barriers to implementation may need to be, at least partially, addressed for such research to occur.

Unfortunately, while these results indicate interventions like Jaspr are promising, there are barriers to more widespread implementation of these types of tools, particularly with regard to payment, as well as regulation and its associated costs. There is not currently a Current Procedural Terminology (CPT) code, which allows for adequate standardizing costs and billing for the use of such tools to payers. A CPT code is needed for more complex delivery platforms to help underwrite the costs associated with a software platform that is HIPAA compliant and integrates into an electronic health record. The lack of CPT code is a significant barrier to widespread adoption, as it is not feasible for each health care system to negotiate its own rate for each individual technology. Moreover, while these interventions are potentially powerful, for problems like suicide, they will likely require at a minimum, an application to the Food and Drug Administration to determine whether oversight is required. If it is, this is an extremely time-consuming, expensive process, the cost of which might need to be incorporated into the cost associated with a CPT code and would ensure that the system is compensated in a way where they can afford the actual cost of a tool like Jaspr. Given that many tools like Jaspr are developed by start-up companies or academic laboratories on shoe-string budgets, models for an effective pathway toward addressing these barriers is needed. Preliminary, real-world data with large samples on how these tools are used by providers and their associated patient outcomes, are an important step in helping provide a rationale for solving these implementation barriers.

### Limitations

Despite the strengths of this study, several limitations are notable. First, the data were gathered in a real-world context with no experimental controls. Without a control condition, blinded assessment, and other experimental controls, it is impossible to know whether decreases in agitation and distress were due to the delivery of evidence-based care via the app itself, the passing of time, or other interventions received in the ED. This finding is, however, consistent with outcomes from our previously published RCT [37]. In addition, a follow-up assessment or information about subsequent hospitalizations would provide additional data on benefits to patients due to Jaspr use. Second, it is impossible to know whether the AE data accurately reflect actual AEs that may have occurred through use of Jaspr or Jaspr-at-Home. While medical personnel and patients could easily report an AE via the app or to staff, it is possible that providers were unaware that an AE occurred (eg, they were not with the patient when it happened; too busy responding to medical emergencies to record an AE) or patients may have failed to report an AE (eg, could not easily discriminate further distress caused by app usage; lacked motivation to record an AE). Third, in addition, there was no assessment of whether app use reduced personal contact time with ED staff (ie, substitution effects) or increased wait times for patients using Jaspr (eg, triaging care to those who had declined). Fourth, since Jaspr records a “completed” event in a binary fashion, it is not possible to identify the number of cases that were near completion. It is possible, for example, that usage and engagement are far greater than captured in the conservative “completion” data. Fifth, there may be specific barriers to engagement and ongoing use that were not assessed in the context of this study. For instance, age could be a factor; it is possible that older adults and others less conversant with digital technologies may struggle to use Jaspr during a period of heightened distress (ie, in the ED), and be less likely to engage in ongoing use post discharge. Sixth, no data on functional changes in patient behavior were captured in this data set. Future studies should seek to capture this data. Seventh, there was no way to reliably determine why patients discontinued Jaspr use (eg, transfer/discharge vs dislike of the app). Eighth, it is also possible that there were ED conditions that impacted implementation that were not captured, but relevant for more widespread adoption (eg, increased ED chaos leading to decreased Jaspr assignment). Ninth, data on the number of users who were eligible but not offered Jaspr, as well as users who were offered but declined to participate was not collected, would provide helpful context to understanding the implementation of Jaspr. Tenth, there is an assumption that stabilization (ie, reductions in distress and agitation) follows delivery of evidence-based practices via the Jaspr app; however, further investigation is needed to determine the mechanisms by which these changes occur. Eleventh, finally the specific data points are limited to a modest number of variables because of prioritizing reduced assessment burden in the context of real-world use, and because of the method of data collection (ie, internal Jaspr data analytics) we do not have data on sample that would provide important information on generalizability and potential sources of bias (eg, patient demographics for subgroup analyses).

### Conclusions

This study of an evidence-based app for individuals with acute suicidal ideation seeking suicide care in the ED supports its safety when integrated into natural workflows, without clinical trial procedural controls. Comprising 10 EDs and 1 inpatient setting across 2 large US-based health care systems, 962 patients with acute suicidal ideation were assigned the Jaspr crisis app as part of their suicide care. At least 942 patients used Jaspr; 489 patients used Jaspr to thoroughly and safely complete an evidence-based crisis stability plan, and another 591 thoroughly and safely completed an evidence-based lethal means assessment. Given the significant shortage of ED medical providers available to deliver robust suicide care, digital technologies like Jaspr may facilitate provision of thorough and life-saving evidence-based suicide care at the point of need. To the extent that Jaspr is designed for, tested with, and safe for use with a highly vulnerable population, this research may also pave the way for similar behavioral health digital interventions for others in need of evidence-based care.

## Appendix

Multimedia Appendix 1 Jaspr Workflow and Screenshots.

## Abbreviations

AE: adverse event
CAMS: Collaborative Assessment and Management of Suicidality
CAU: care-as-usual
CPT: Current Procedural Terminology
ED: emergency department
HIPAA: Health Insurance Portability and Accountability Act
IRB: institutional review board
RCT: randomized controlled trial
SSI: Suicide Status Interview
TJC: The Joint Commission

## Data Availability

The data sets generated during and analyzed during this study are available from the corresponding author on reasonable request.
