# Supplementary material for: A Suicide Prevention Digital Technology for Individuals Experiencing an Acute Suicide Crisis in Emergency Departments: Naturalistic Observational Study of Real-World Acceptability, Feasibility, and Safety
Source: JMIR Form Res. 2024 Sep 16;8:e52293. doi: 10.2196/52293 (PMC11443178; doi:10.2196/52293)
Supplement: Multimedia Appendix 1 [file formative_v8i1e52293_app1.pdf]

Jaspr Health

Preventing Suicide by Intervening in the Moments that Matter

"…it's one of the best experiences that I've had ever in a hospital."

- a patient in the ED using Jaspr

Transforming Wait Times to Empower Patients and Advance Care

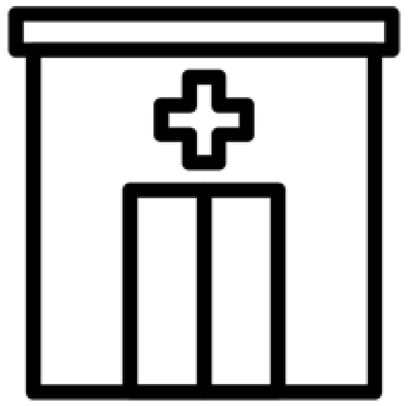

Patient comes into care setting and identified at risk for suicide

"There's an incredible amount of waiting and an incredible amount of trauma being a mental health patient in the hospital. You're locked into a room, or your handcuffed; you're in restraints in the hallway. You wait for hours and hours not knowing what's going to happen. When you do get to talk to people, it's strictly triage: Where are we going to put them? There's no direct interventions or skill-building or counseling. It's just Assess-and-Go."

Multiple Suicide Attempt Survivor,

Without Jaspr

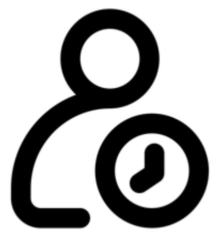

Patient is left alone without resources while waiting for care.

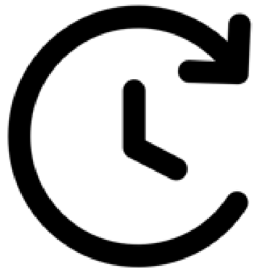

Patient stays in high distress, ruminates on problems; feels hopeless, isolated; patient frightened and doesn't know what to expect.

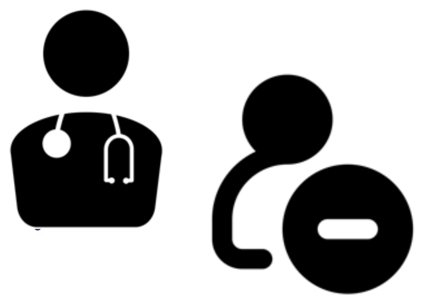

Provider conducts assessment and seeks a psychiatric inpatient bed to transfer patient – or boards in ED.

With Jaspr

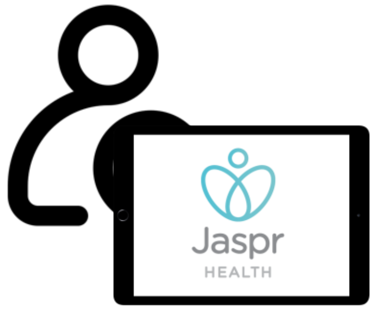

Patient receives evidence-based suicide care via Jaspr tablet while waiting for their provider.

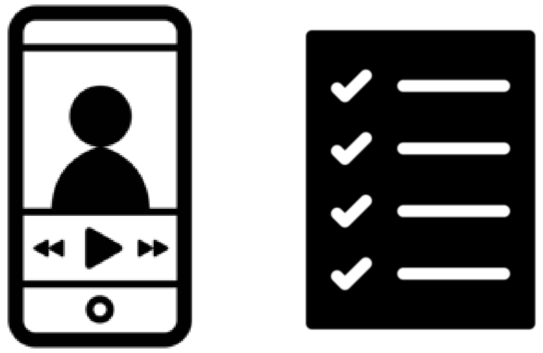

Patient feels calmer, more hopeful, learned new skills to cope better; feels less alone from sharing their story.

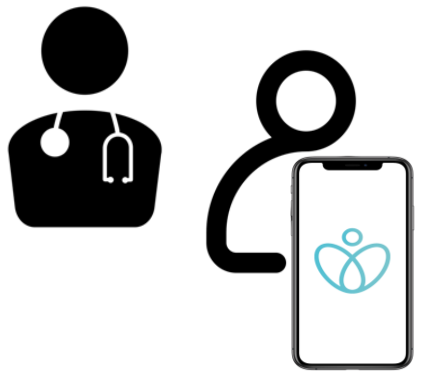

Provider reviews Jaspr assessment, identifies key areas for interview; documentation is automated.

Patient receives Jaspr-at-Home companion app to reduce suicide risk and readmission post-discharge.

Patient-Clinician Workflow

SUPPORTING EVIDENCE-BASED CARE AT SCALE

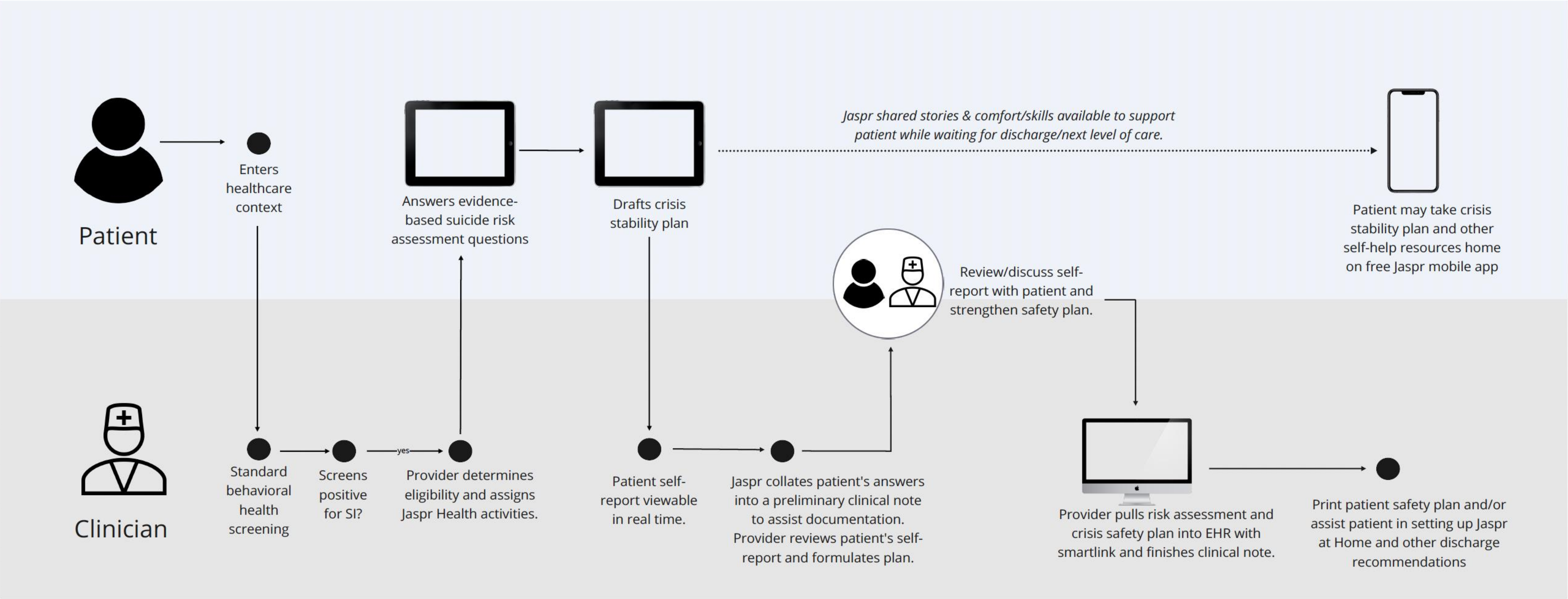

Clinician time optimized, regulatory compliance improved

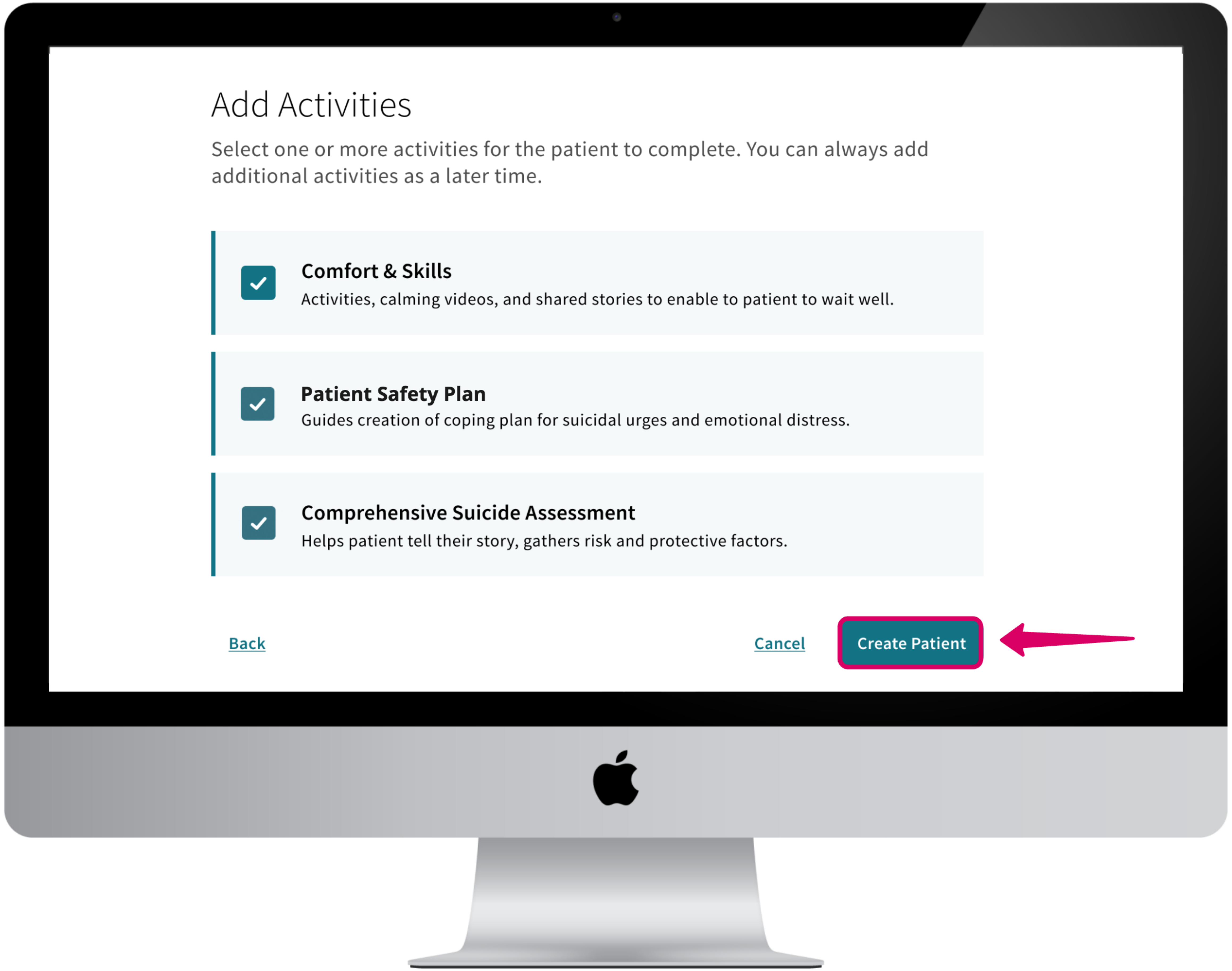

*** Note – these are examples of how Jaspr *may* appear in your Healthcare system's medical chart. These are customized to your site as part of the planning stage of implementation.

Patient Session Begins with Onboarding

Patient introduced to Jaspr with a compassionate welcome video

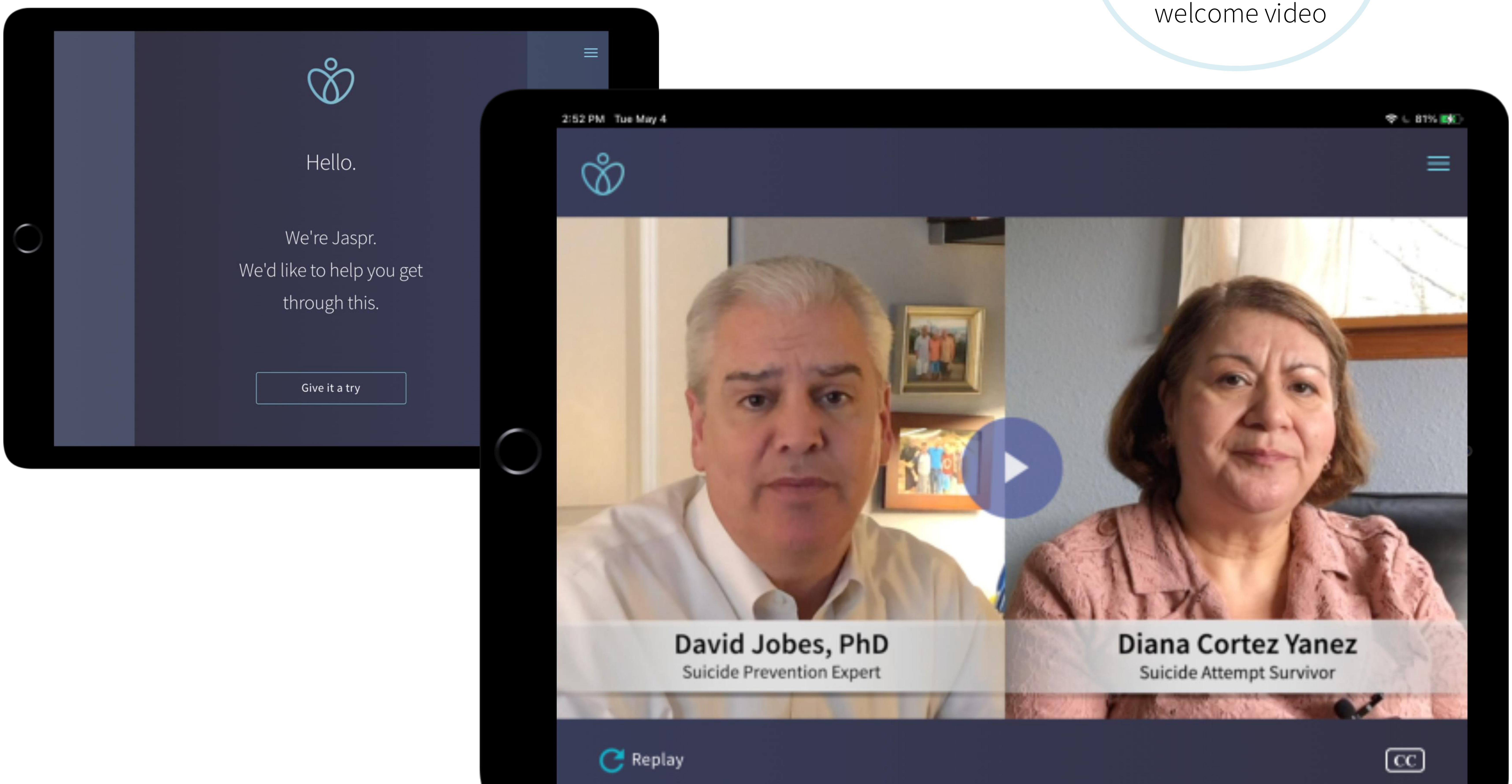

Introduction to Jaz

Patient introduced to automated assistant and begins to answer baseline metrics.

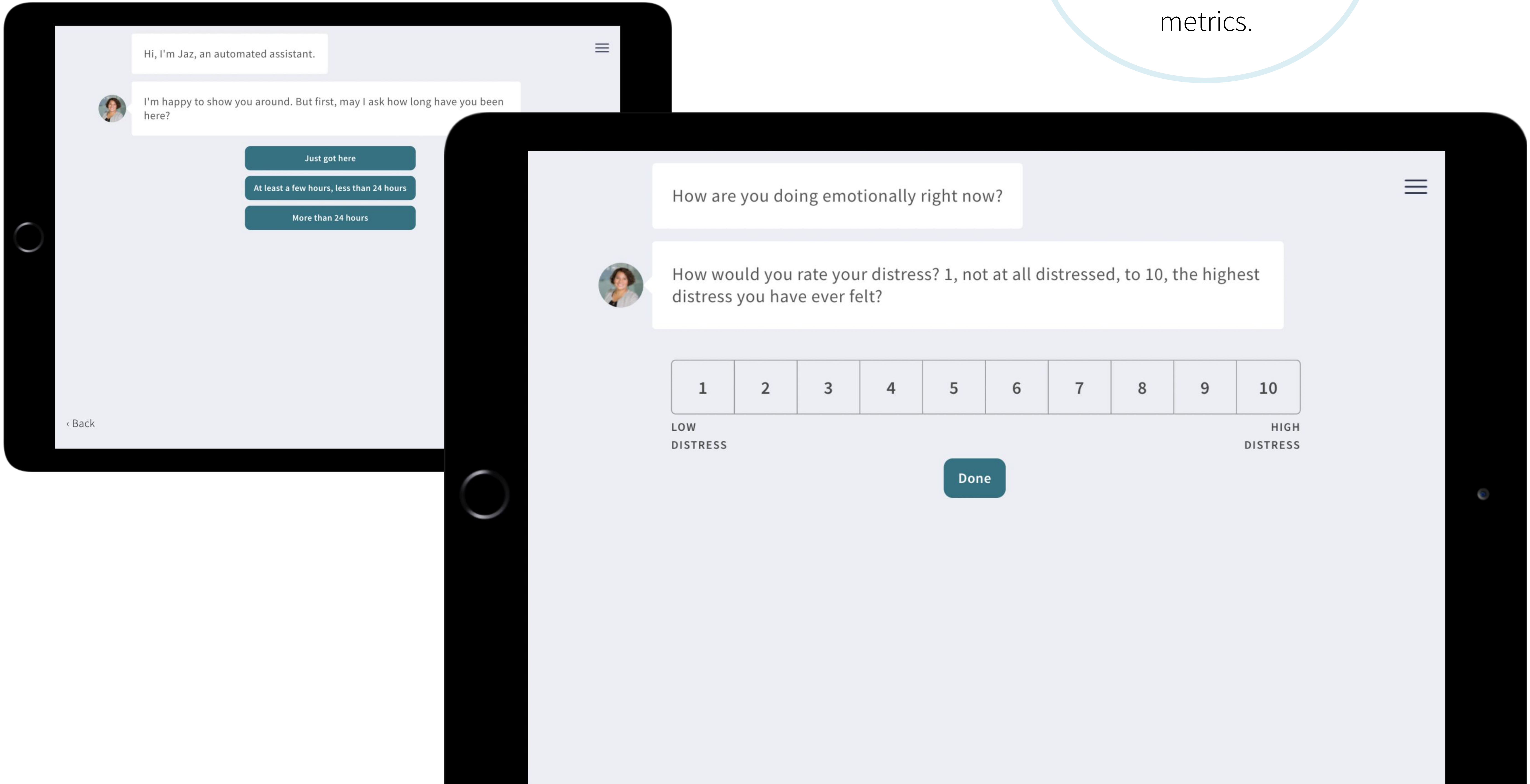

Jaspr Health – Content Exploration

Patient is guided through the following activities:

- Suicide Risk Assessment
- Crisis Safety Plan
- Lethal Means Counseling
- Comfort/Skills Videos
- Shared Stories Videos

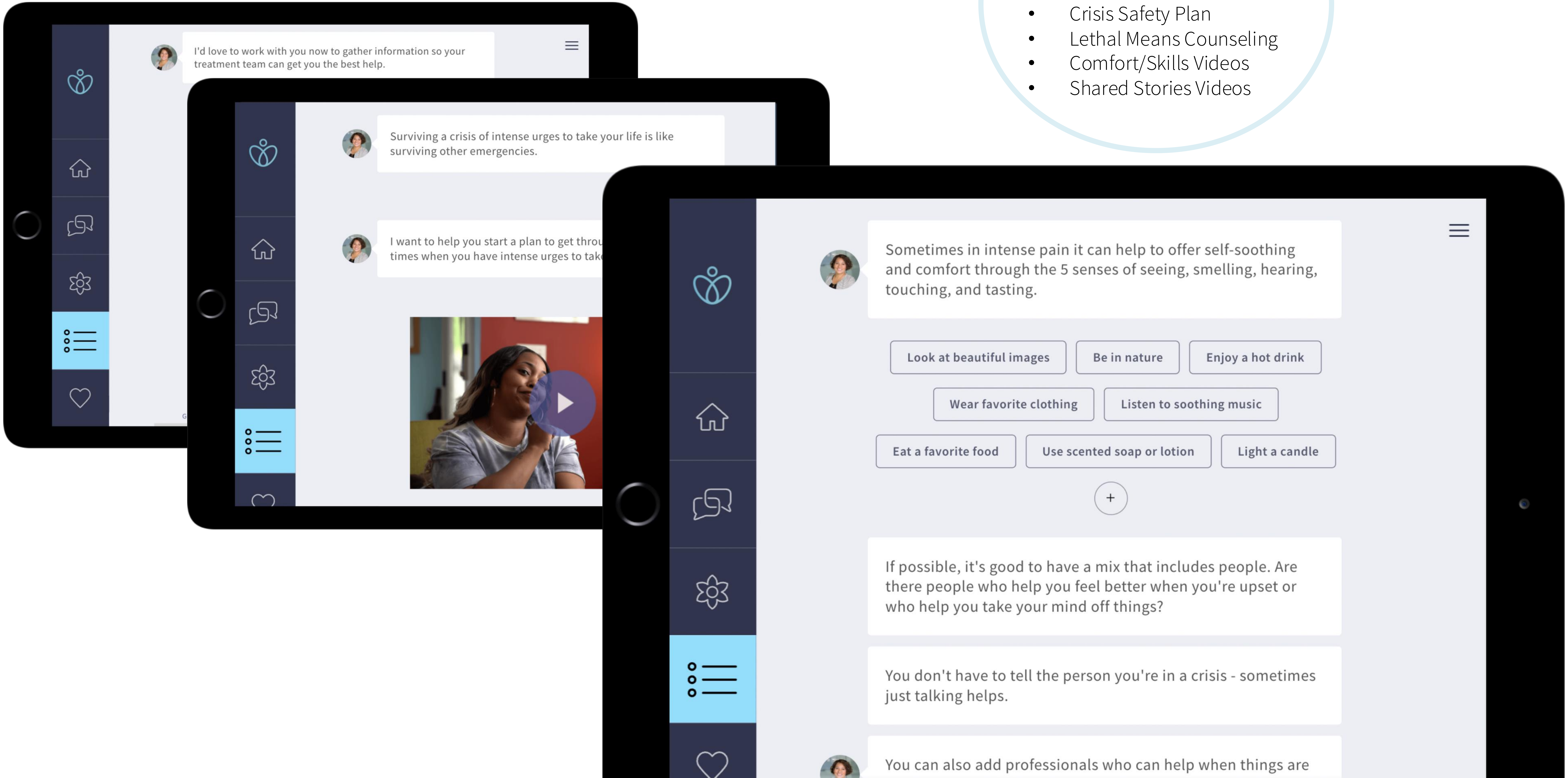

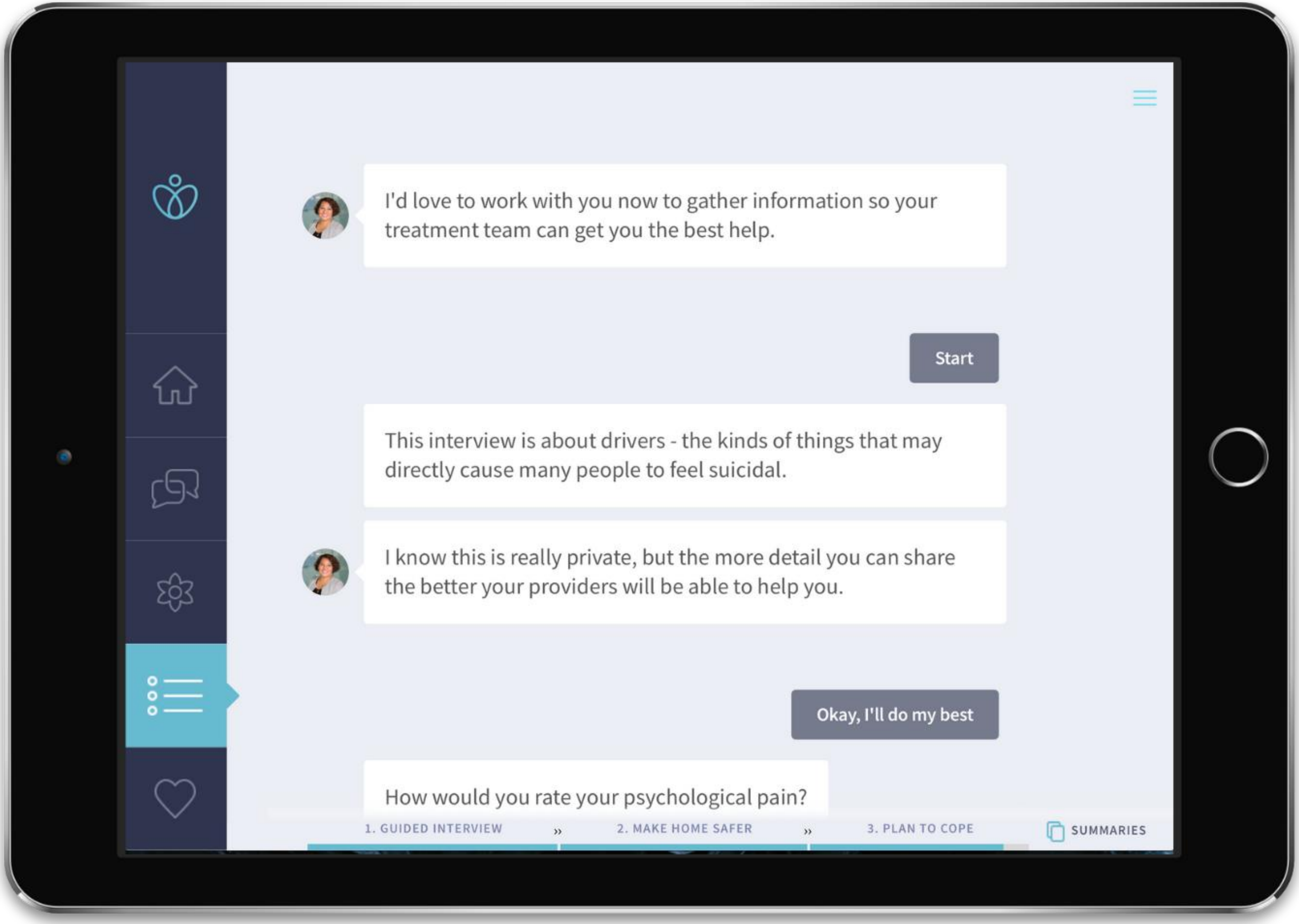
I'd love to work with you now to gather information so your treatment team can get you the best help.
Start
This interview is about drivers - the kinds of things that may directly cause many people to feel suicidal.
I know this is really private, but the more detail you can share the better your providers will be able to help you.
Okay, I'll do my best
How would you rate your psychological pain?
1. GUIDED INTERVIEW
2. MAKE HOME SAFER
3. PLAN TO COPE
SUMMARIES

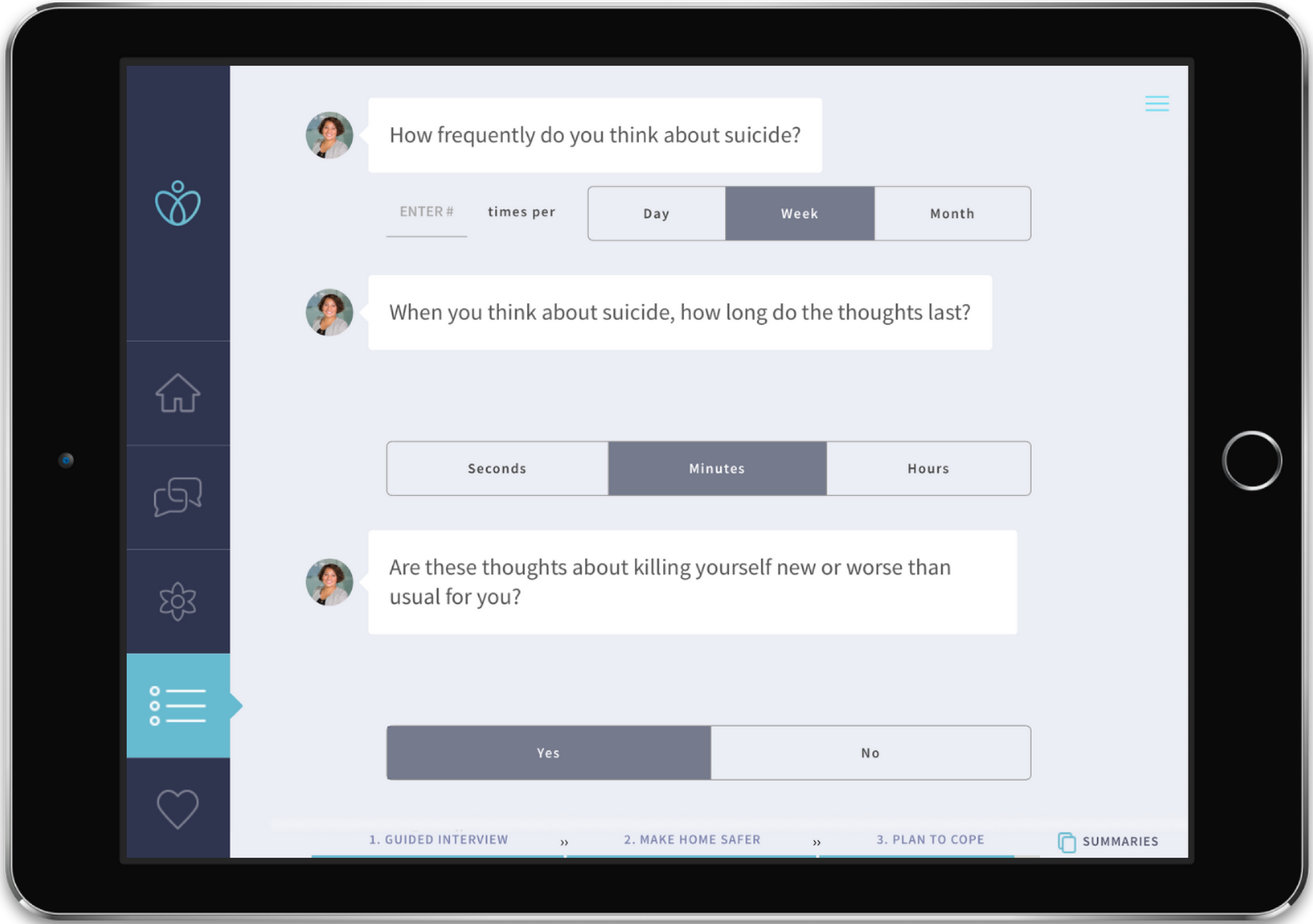
How frequently do you think about suicide?
ENTER #
times per
Day
Week
Month
When you think about suicide, how long do the thoughts last?
Seconds
Minutes
Hours
Are these thoughts about killing yourself new or worse than usual for you?
Yes
No
1. GUIDED INTERVIEW
2. MAKE HOME SAFER
3. PLAN TO COPE
SUMMARIES

Therapeutic Video Content

Patient may explore a variety of video content to "wait well". Videos include guided DBT skills and compassionate stories from those with lived experiences.

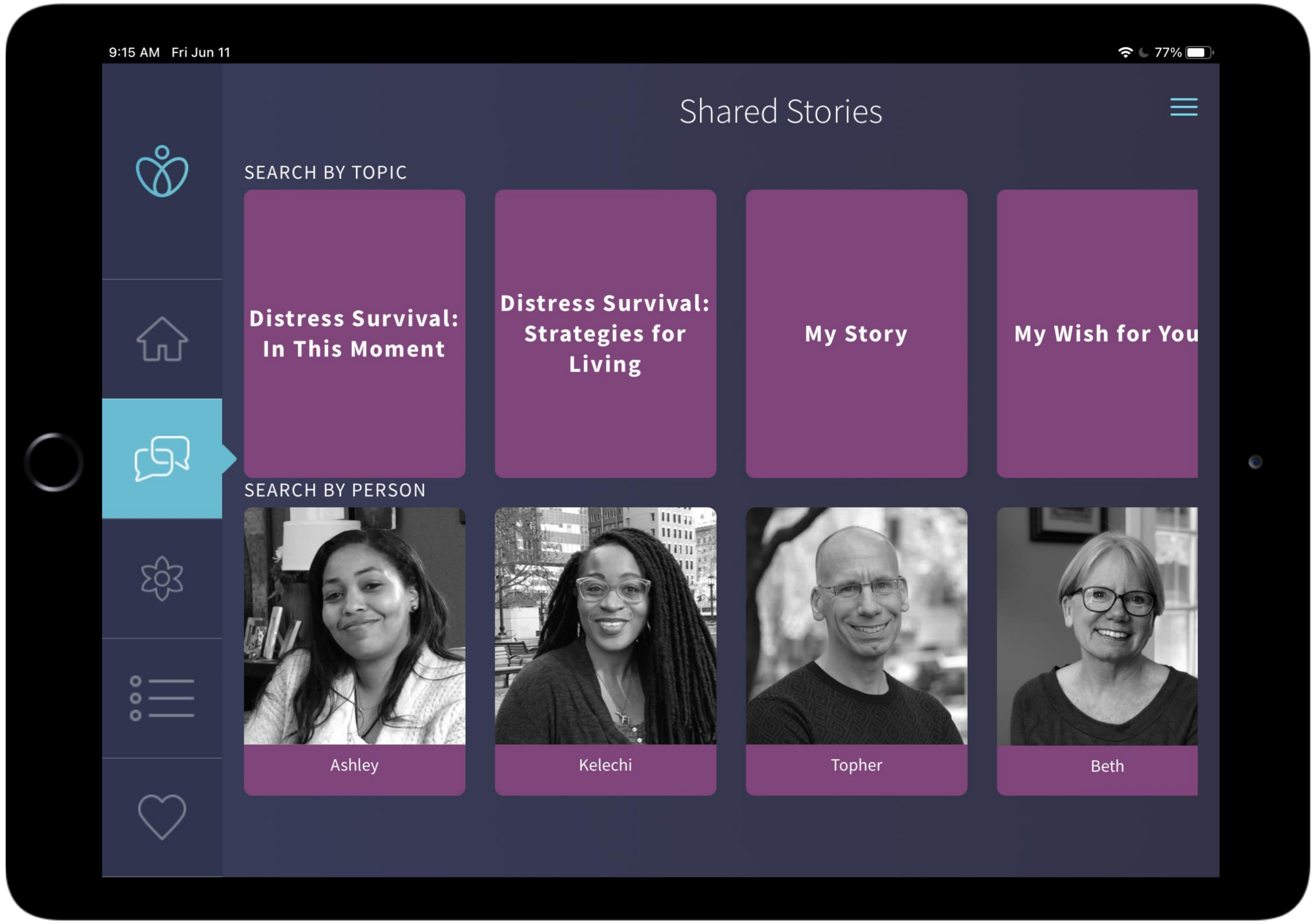

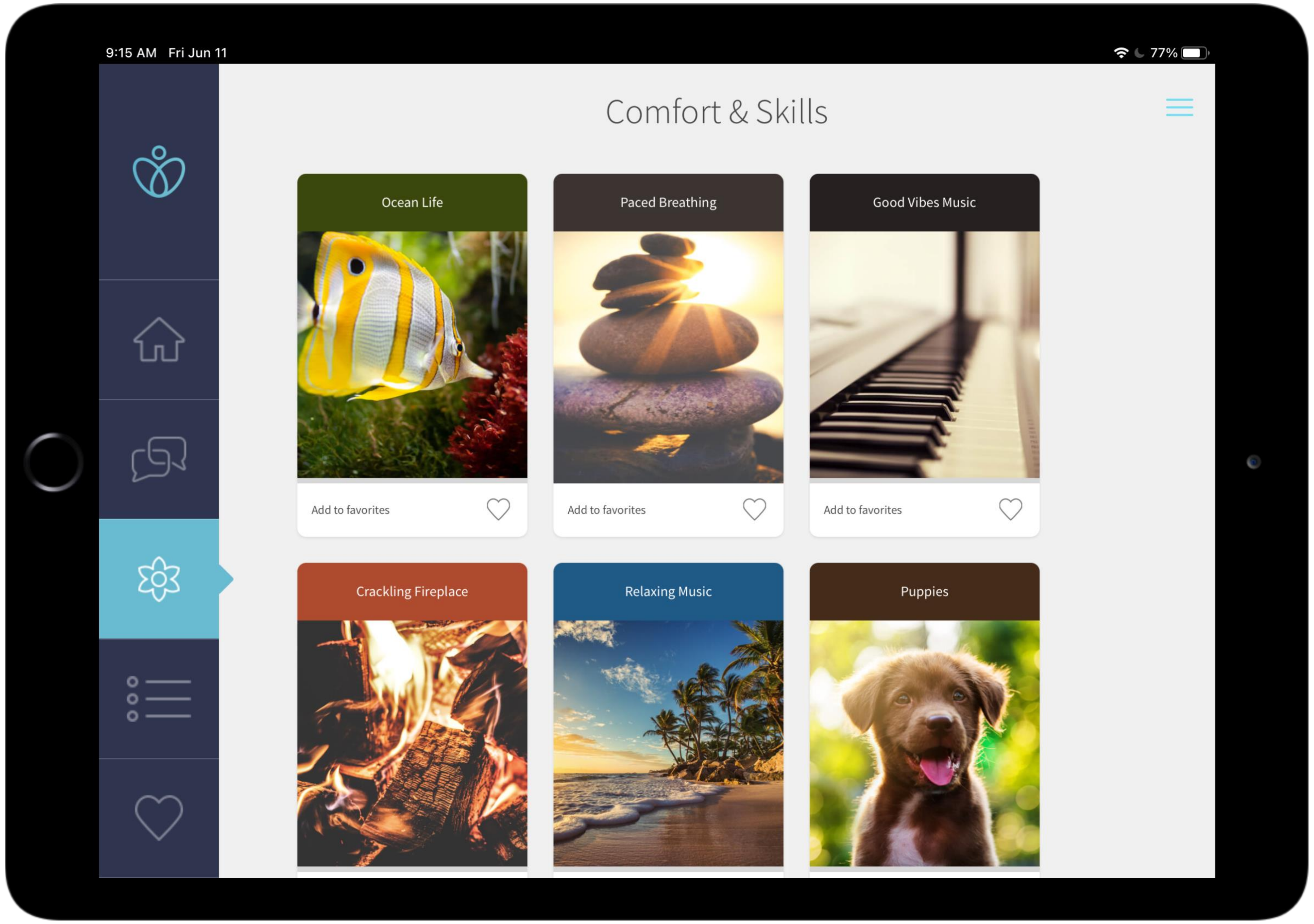

Content for Teen & Families

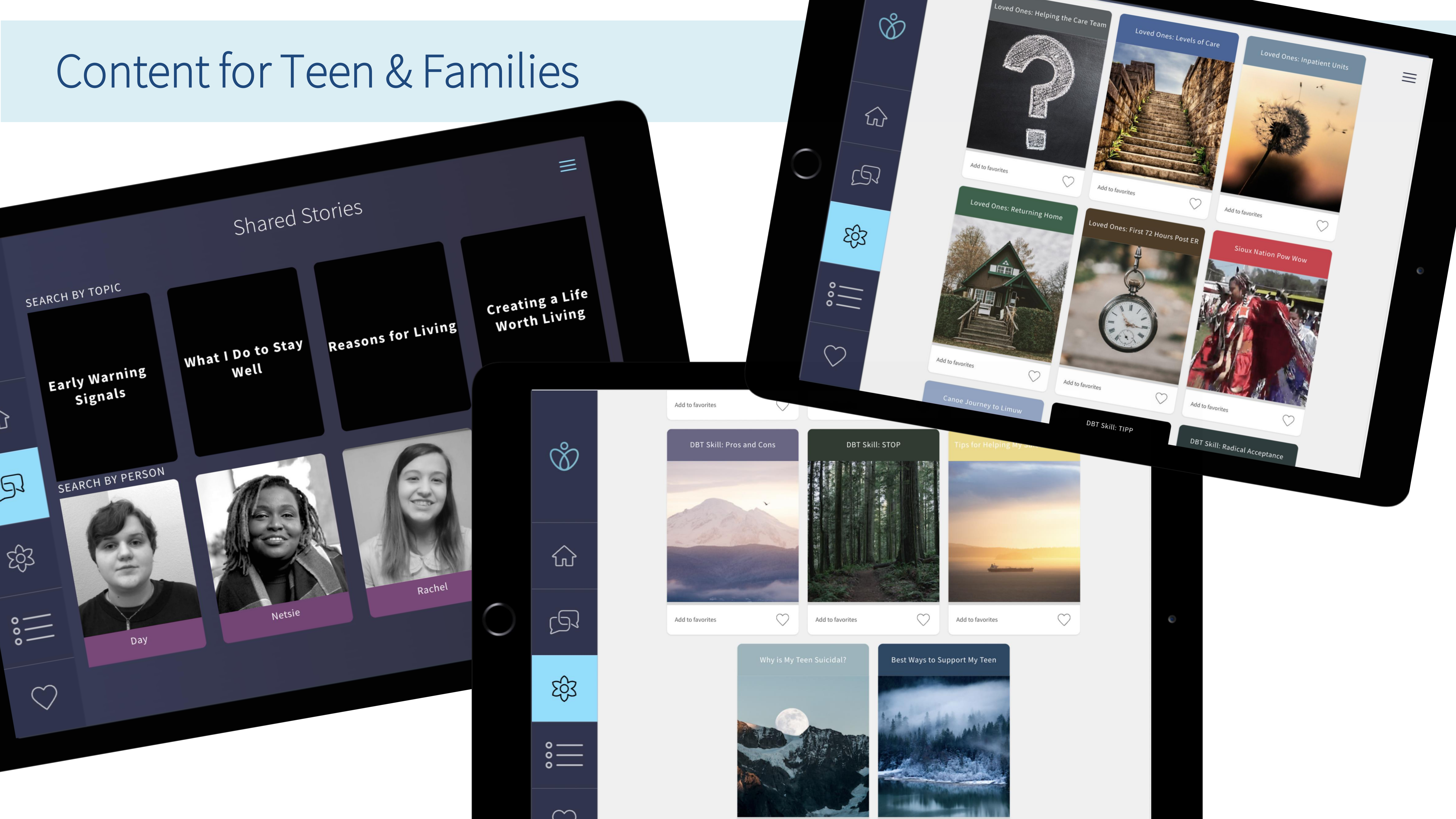

Shared Stories & Skills

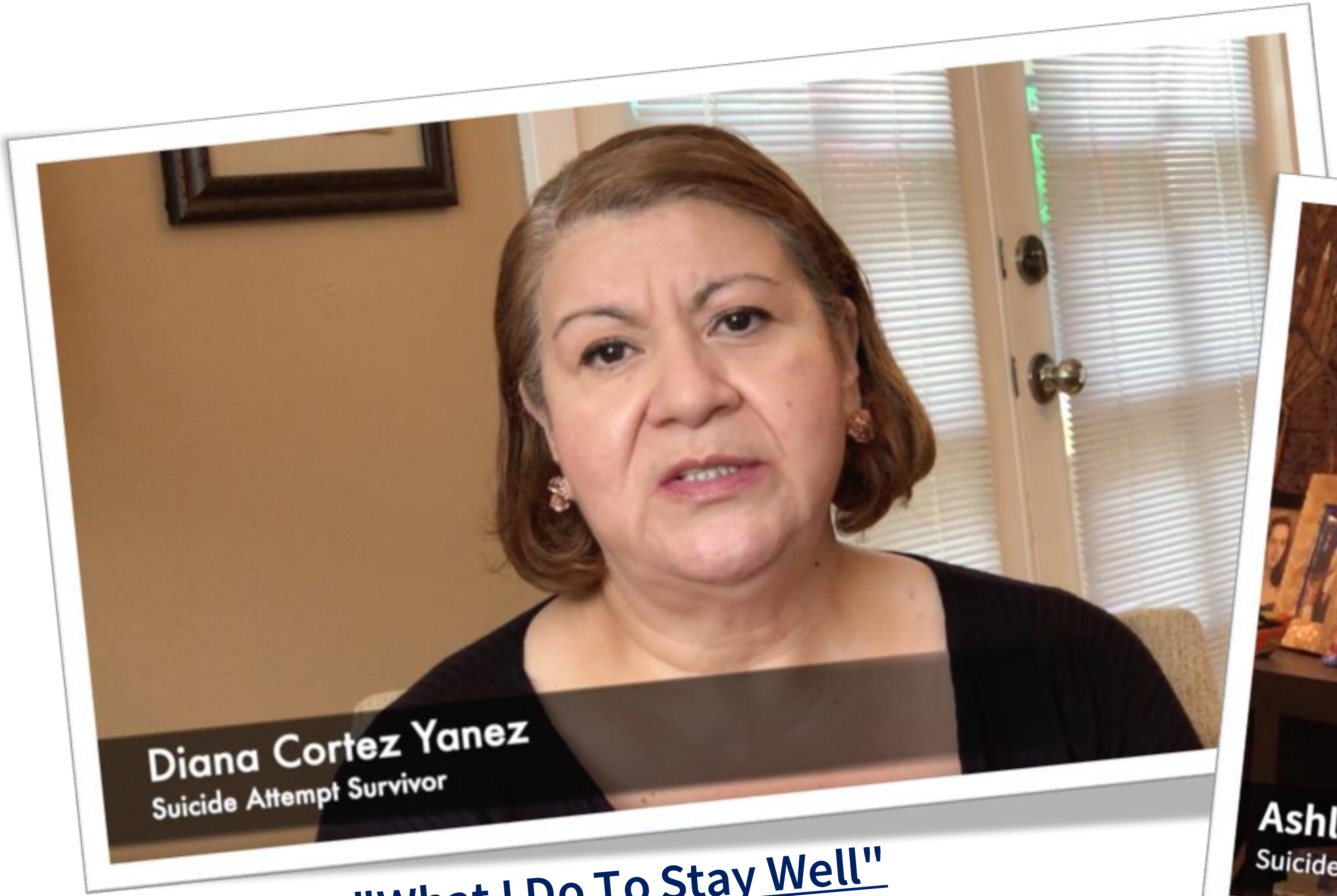

"What I Do To Stay Well"

"My Wish For You"

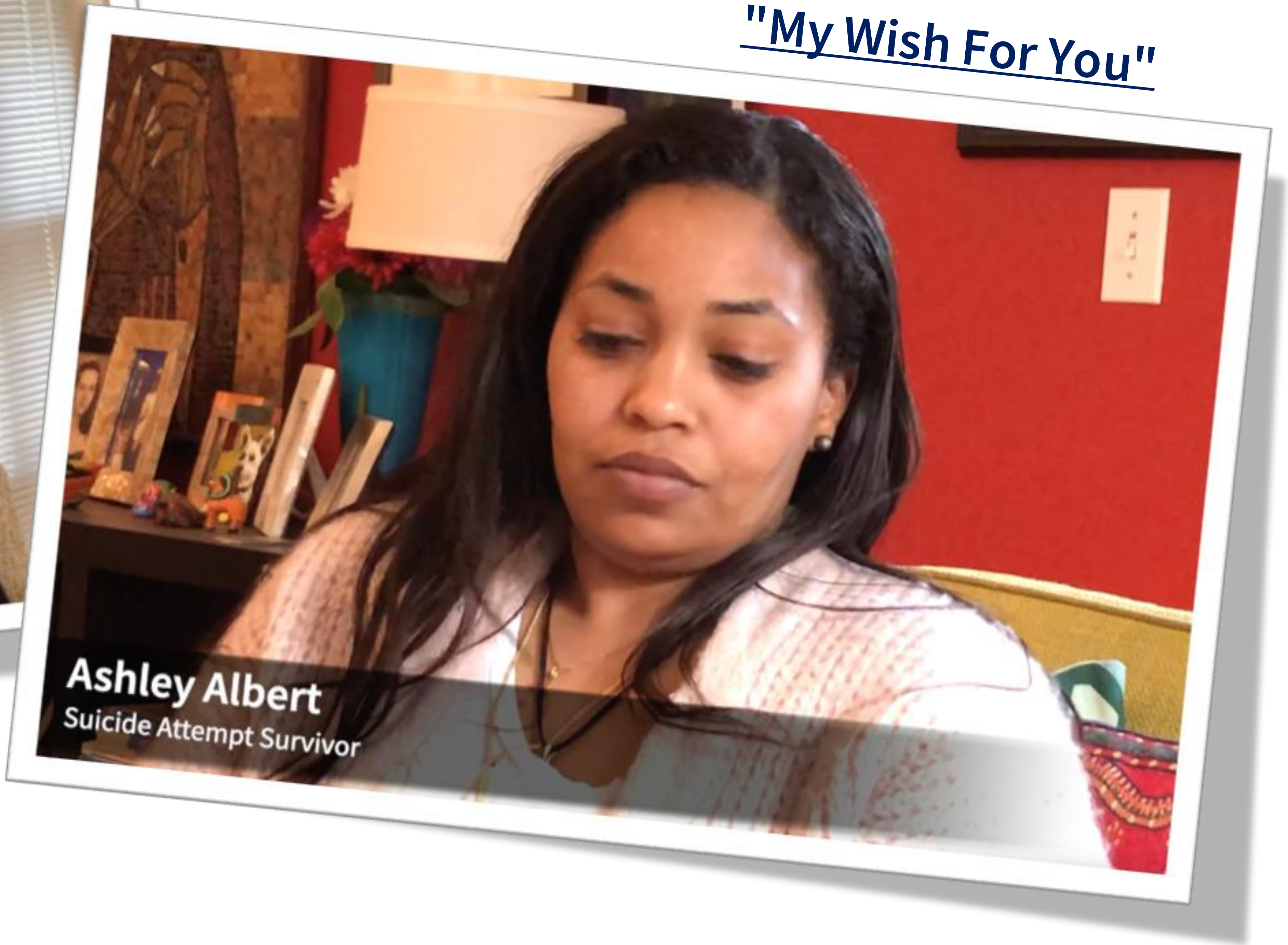

People with Lived Experience inform all aspects of Jaspr Health’s product and research

- Share wisdom and messages of hope
- Teach coping life skills
- Provide psychoeducation
- Reduce stigma associated with struggling with suicidality and behavioral health problems
- Provide research insights

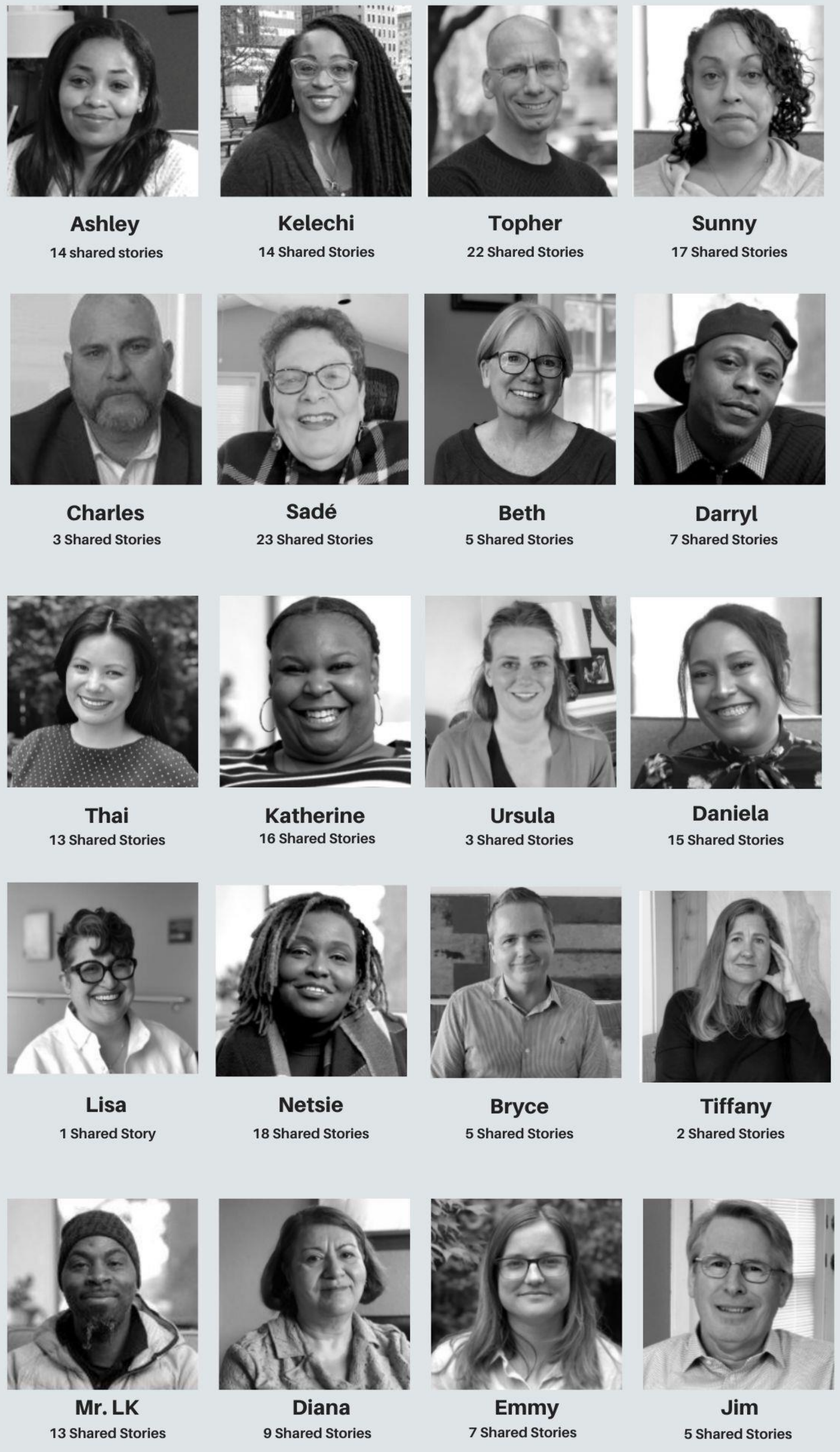

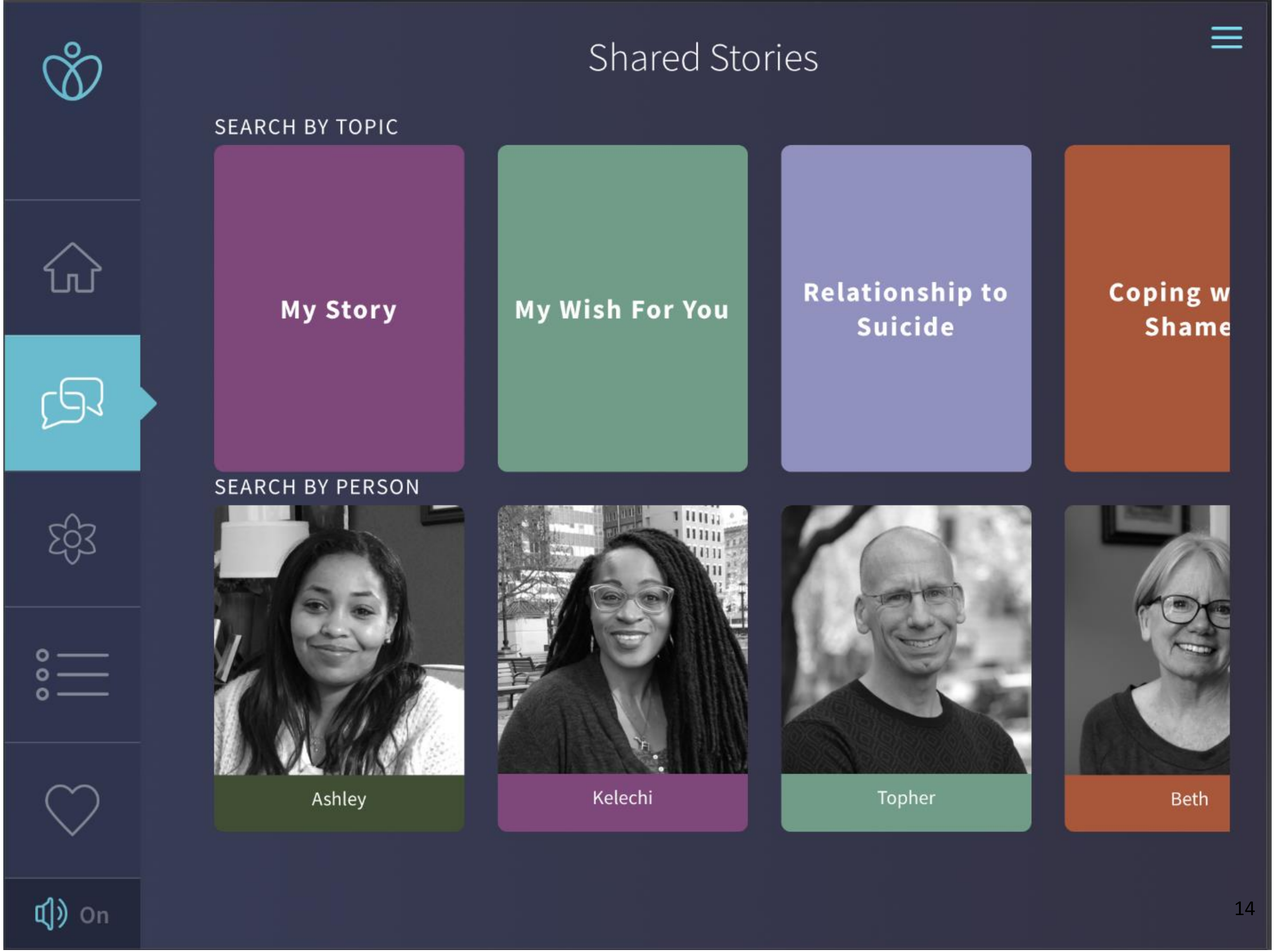
Shared Stories
SEARCH BY TOPIC
My Story
My Wish For You
Relationship to Suicide
SEARCH BY PERSON
Ashley
Kelechi
Topher
Beth
On

Takeway Kit – Crisis Safety Plan

The patient can find their saved content and crisis stability plan in their takeway kit.

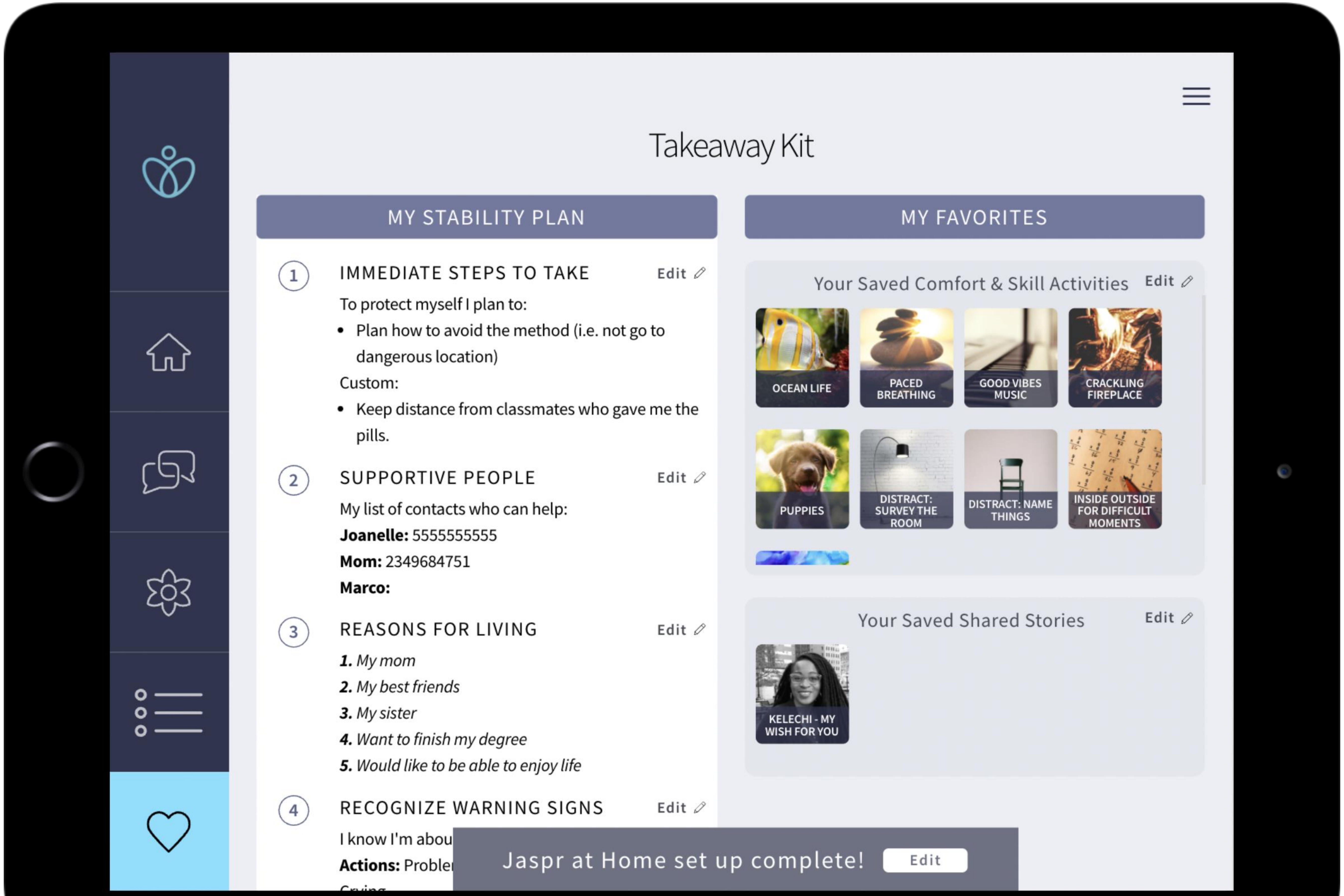

Jaspr-at-Home

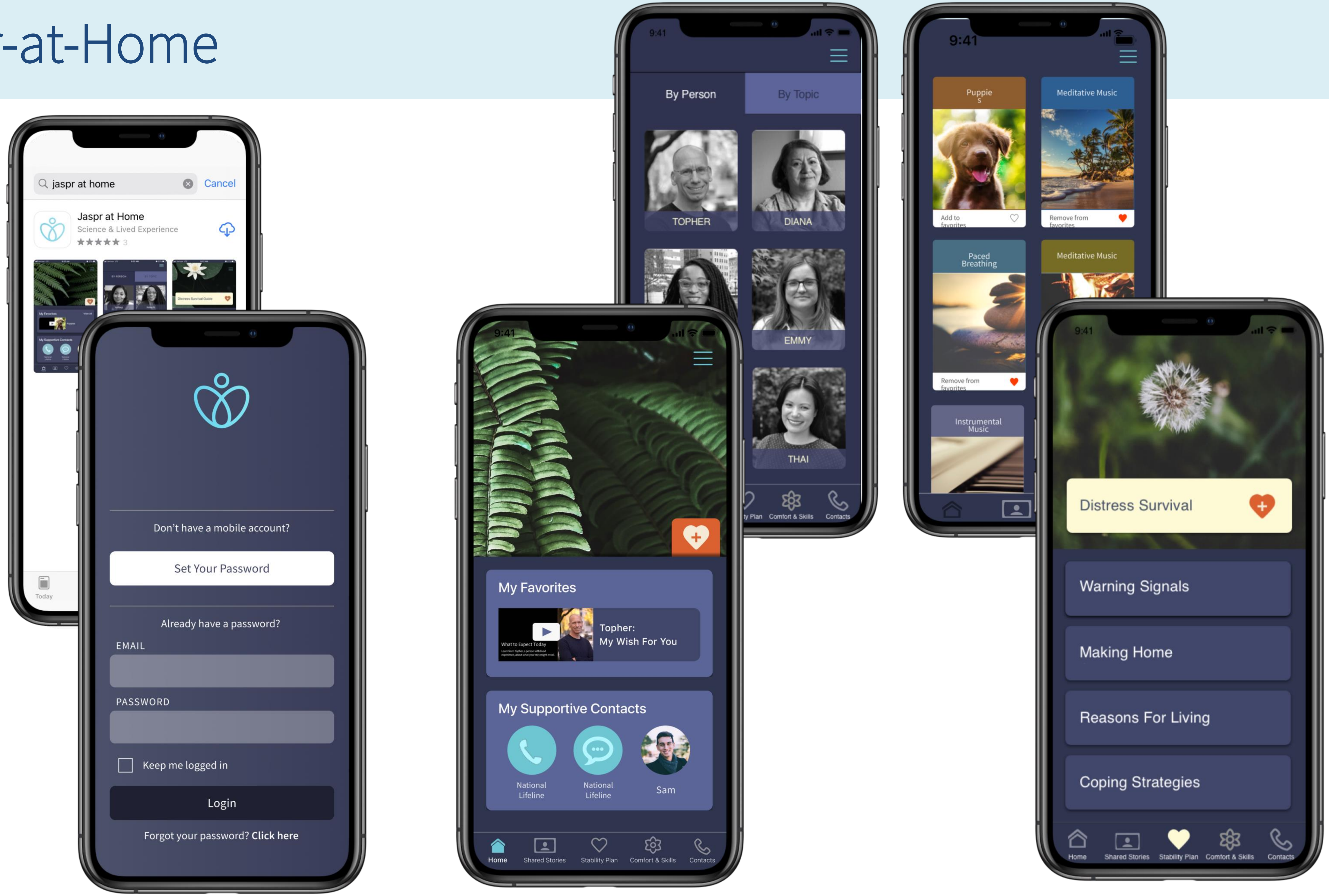

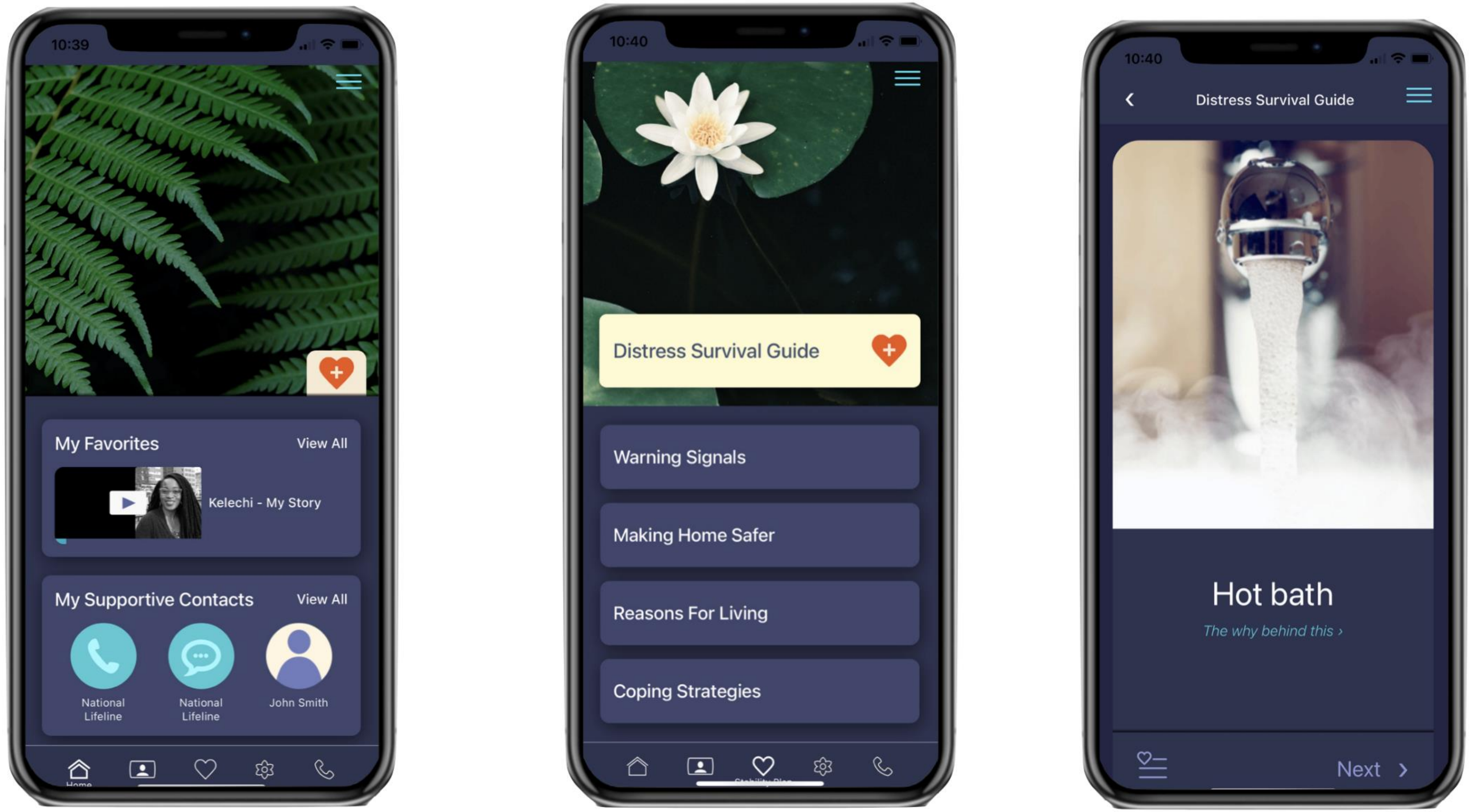

Crisis Response Plan available after acute care with step-by-step ‘playlist’ of coping strategies and PLE messages of hope.

Review patient's progress and self-report in real time.

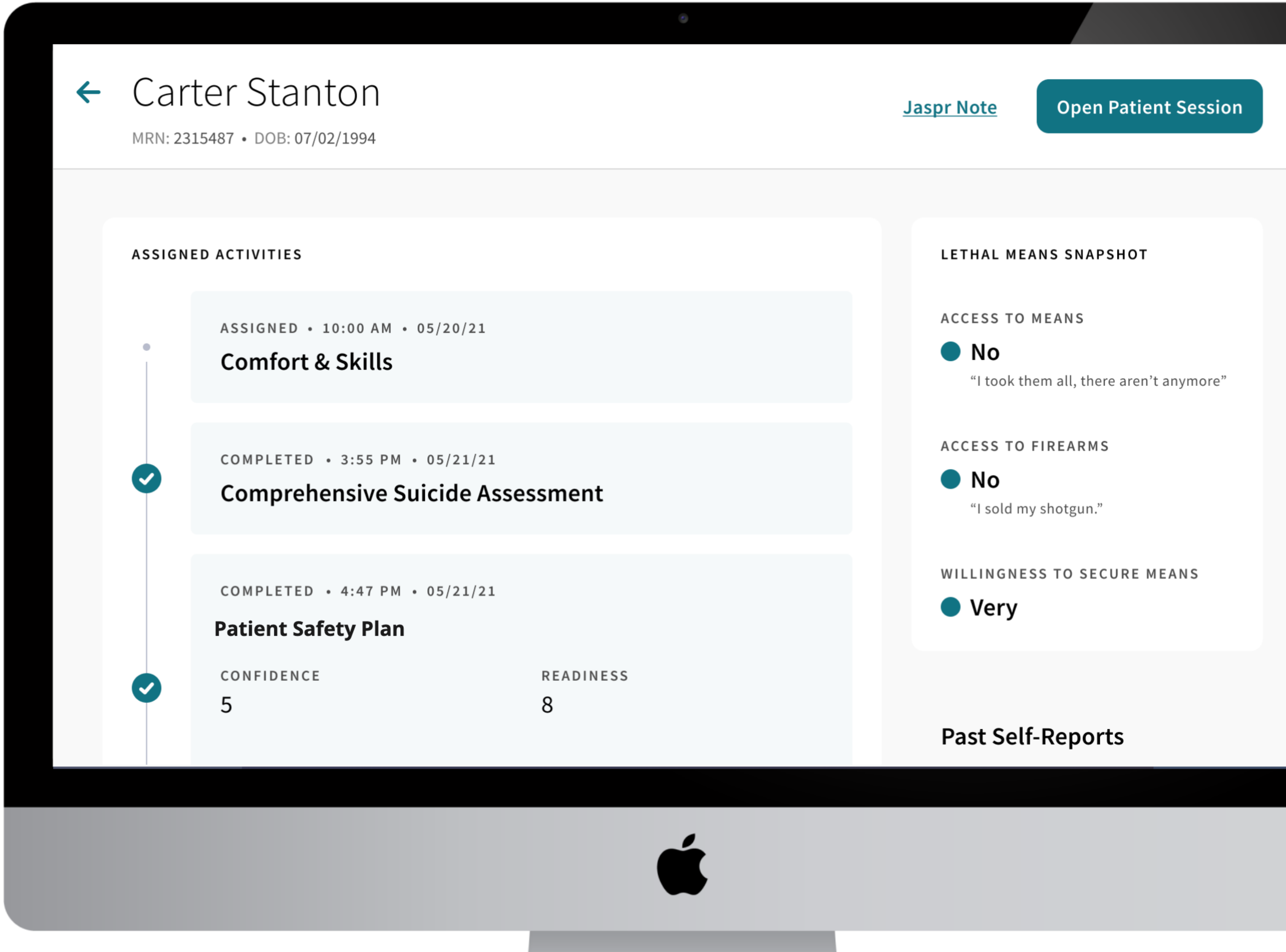

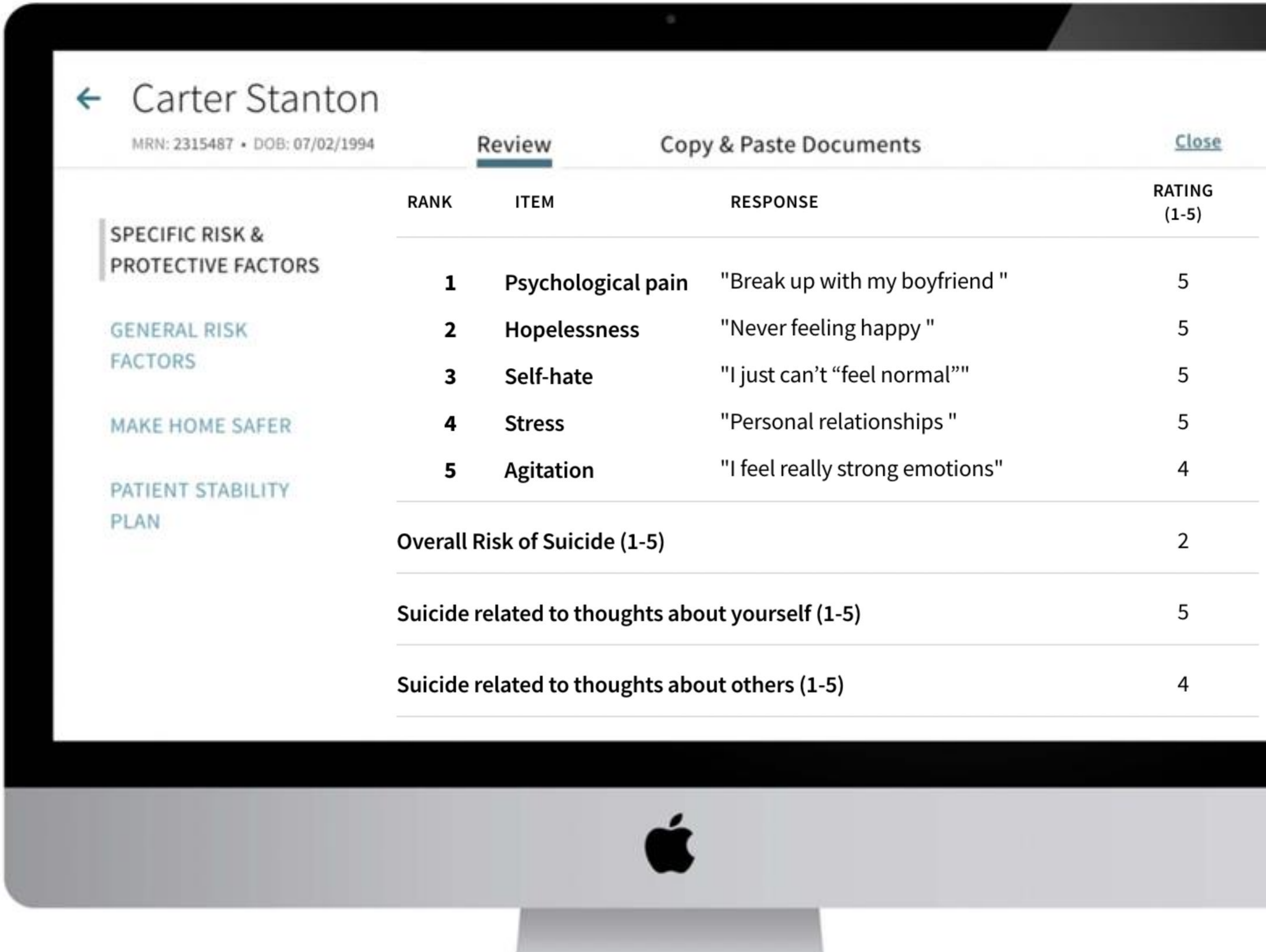
Carter Stanton
MRN: 2315487 • DOB: 07/02/1994
Review
Copy & Paste Documents
Close
SPECIFIC RISK & PROTECTIVE FACTORS
GENERAL RISK FACTORS
MAKE HOME SAFER
PATIENT STABILITY PLAN
RANK
ITEM
RESPONSE
RATING (1-5)
1 Psychological pain "Break up with my boyfriend " 5
2 Hopelessness "Never feeling happy " 5
3 Self-hate "I just can’t “feel normal”" 5
4 Stress "Personal relationships " 5
5 Agitation "I feel really strong emotions" 4
Overall Risk of Suicide (1-5) 2
Suicide related to thoughts about yourself (1-5) 5
Suicide related to thoughts about others (1-5) 4

Thank You

Contact Info:

Linda Dimeff, PhD
Linda.Dimeff@jasprhealth.com
